# Targeted Gene Knockin in Porcine Somatic Cells Using CRISPR/Cas Ribonucleoproteins

**DOI:** 10.3390/ijms17060810

**Published:** 2016-05-26

**Authors:** Ki-Eun Park, Chi-Hun Park, Anne Powell, Jessica Martin, David M. Donovan, Bhanu P. Telugu

**Affiliations:** 1 Department of Animal and Avian Sciences, University of Maryland, College Park, MD 20742, USA; kepark@umd.edu (K.-E.P.); chpark@umd.edu (C.-H.P.); jjm11393@umd.edu (J.M.); 2 Animal Bioscience and Biotechnology Laboratory, USDA-ARS, Beltsville, MD 20705, USA; anne.powell@ars.usda.gov (A.P.); david.donovan@ars.usda.gov (D.M.D.); 3 Livestock Genomix, Reisterstown, MD 21136, USA

**Keywords:** CRISPR/Cas, SCNT, cloning, knockin, TARGATT pig, transegenic

## Abstract

The pig is an ideal large animal model for genetic engineering applications. A relatively short gestation interval and large litter size makes the pig a conducive model for generating and propagating genetic modifications. The domestic pig also shares close similarity in anatomy, physiology, size, and life expectancy, making it an ideal animal for modeling human diseases. Often, however, the technical difficulties in generating desired genetic modifications such as targeted knockin of short stretches of sequences or transgenes have impeded progress in this field. In this study, we have investigated and compared the relative efficiency of CRISPR/Cas ribonucleoproteins in engineering targeted knockin of pseudo attP sites downstream of a ubiquitously expressed *COL1A* gene in porcine somatic cells and generated live fetuses by somatic cell nuclear transfer (SCNT). By leveraging these knockin pseudo attP sites, we have demonstrated subsequent phiC31 integrase mediated integration of green fluorescent protein (GFP) transgene into the site. This work for the first time created an optimized protocol for CRISPR/Cas mediated knockin in porcine somatic cells, while simultaneously creating a stable platform for future transgene integration and generating transgenic animals.

## 1. Introduction

The pig is an important dual purpose animal model for agriculture and biomedical applications. With an increasing global population and an increased demand for animal protein, domesticated animals such as pigs are critical for tackling the emerging global food security crisis. Unlike domestic ruminants, the pig has a short gestational interval (114 days), is a litter bearing animal carrying an average of 14 piglets in one pregnancy, and in commercial setting can give rise to three pregnancies in one year. These attributes make it not only a valuable model for agriculture but also for genetic engineering applications, where relatively short pregnancy and large litter size is preferred for generating and propagating genetically modified animals. From a biomedical standpoint, there is an increased awareness among the biomedical community that mouse models cannot meet the complete spectrum of biomedical needs, and an alternative animal model such as the pig is required to meet the shortcomings of the mouse model [1,2,3,4,5].

In domestic pigs, the preferred means for generating genetically engineered animals is somatic cell nuclear transfer (SCNT), where somatic cells typically fetal fibroblasts are modified to include the intended genetic modification and used as nuclear donors for generating genetically modified offspring. The most common genetic modification is transgenesis, where the transgene of interest is introduced into somatic cells and selected for stable integration of the transgene prior to SCNT. However, random integration of transgenes suffers from potential limitations such as insertional mutagenesis (the transgene inserts into an existing gene potentially disrupting the endogenous gene’s expression or function), lack of control over transgene copy number, silencing or aberrant expression of transgenes in non-target tissues based on the site of integration (positional variegation), random assortment and segregation in subsequent generations, to name a few [6]. In this regard, inserting the transgenes into a specific locus by gene targeting (knockin) is preferable to avoid the concerns outlined above. However, homologous recombination mediated gene targeting events suffer from poor efficiencies in somatic cells (1 in 10^6^–10^7^ cells). An additional limitation with the use of most commonly used somatic cells (fetal fibroblasts) is their limited viability in culture for screening recombinants. Site specific nucleases or genome editors such as ZFNs (zinc finger nucleases), TALENs (transcription activator-like effector nucleases), and CRISPR (clustered regulated interspaced short palindromic repeat) and CRISPR-associated (Cas) nuclease system (CRISPR/Cas) that engineer a double strand break (DSB) at the target site and promote gene targeting or homologous recombination can improve efficiencies by nearly 1000 fold and thus could offer a solution [7].

Among the available editors, the CRISPR/Cas system has emerged as a tool of choice in most laboratories because of the ease of design, assembly, delivery and a high degree of reliable gene modifications. In pigs and other domestic animals, the CRISPR/Cas system has been employed successfully for the generation of edited animals [8,9,10,11,12,13]. In these studies, a mammalian codon optimized Type III Cas9 from *Streptococcus pyogenes* alongside a chimeric synthetic single-guide RNA (sgRNA) [14] containing Cas9 binding sites and a 20 nt guide sequence specific to the target site has been used to introduce DSBs [8,10,11,12,15]. The DSBs generated by CRISPRs (and other editors) activate endogenous DNA repair pathways that include a predominant error prone non-homologous end joining (NHEJ) or high fidelity homology directed repair (HDR) pathway. For generating gene ablation models, NHEJ is the preferred pathway, whereas for introducing gene knockins and point mutations, the HDR pathway is preferred. In order to achieve HDR at high frequencies, we sought to test several published options that include: the use of a single stranded oligonucleotide as a targeting template, small molecule inhibitors of DNA ligase IV such as SCR7 [16,17], transient incubation of cells at low temperatures [18,19], and finally, the choice of CRISPR reagents. CRISPR reagents can be delivered as DNA expression vectors, RNA preparations, and most recently a ribonucleoprotein complex of Cas9 protein and sgRNA. However, there are no published studies in porcine systems so far that have outlined or examined these different reagents in a head to head comparison. In the current study, we sought to demonstrate that it is possible to create site specific knockins of short DNA sequences, specifically pseudo attP sites (50 bp) downstream of a ubiquitously expressed *COL1A* gene, permitting subsequent phiC31 integrase-mediated introduction of functional transgenes. Serine integrases such as phiC31 integrase cause recombination between two 50 bp recognition sequences attP and attB sites, respectively, and integrate the transgene into the knockin site [20,21]. When such integration takes place site-specifically downstream of a ubiquitously expressed gene, the transgene is expected to be protected from aberrant silencing and positional variegation as outlined above. We correctly hypothesized that a pre-complexed Cas9 protein and sgRNA would be effective in engineering DSBs and facilitating knockin, and that the pseudo attP sites would permit future integration of transgenes into the sites.

## 2. Results

### 2.1. Targeted Knockin of Pseudo-attP Sites Downstream of Porcine COL1A Gene in Somatic Cells Using CRISPR/Cas System

#### 2.1.1. Influence of Temperature on Colony Formation and HDR Outcome

**Figure 1:**
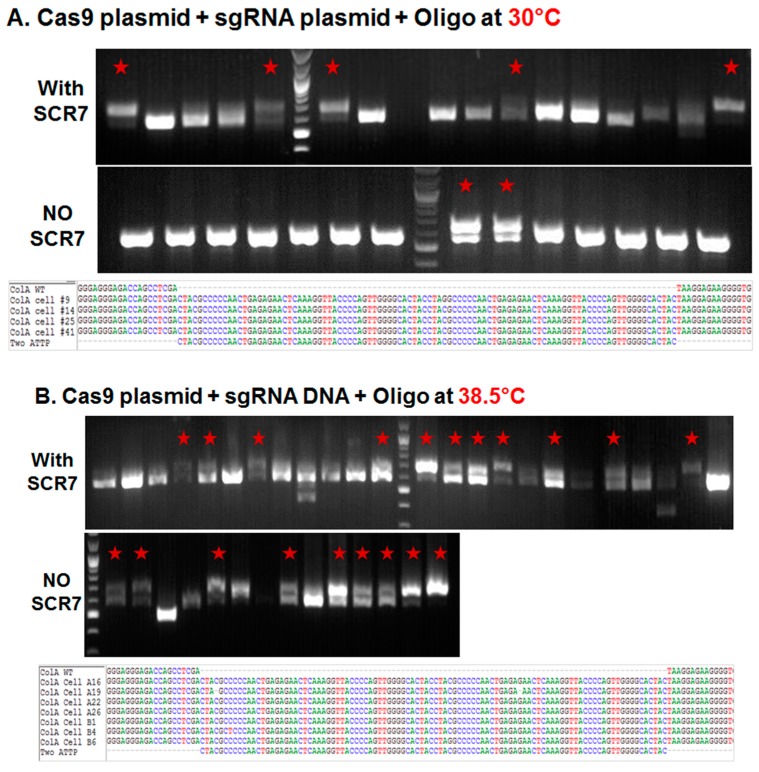

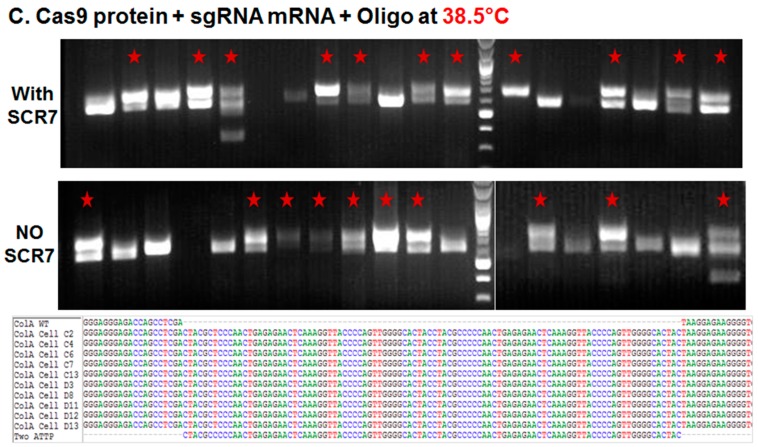
Targeted knockin of two pseudo attP sites (100 bp) downstream of the porcine *COL1A* locus. (**A**–**C**) Agarose gel electrophoresis analysis of amplicons generated by primers bordering the *COL1A* targeting site. PCR amplicons of representative colonies derived from plasmid transfection and transient incubation at 30 °C (**A**) or 38.5 °C (**B**); and ribonucleoprotein (**C**) transfection and culture at 38.5 °C. In each panel, representative colonies showing a new band with predicted shift of 100 bp on the agarose gel produced by successful targeting and introgression of the two pseudo attP sites is marked by a red star. In each panel, the top row represents knockin in the presence of SCR7, the middle row without SCR7 and the bottom row represents Sanger sequencing of the amplicon. Multiple sequence alignment of wild type (WT) *COL1A* gene and two pseudo attP knockin sites are shown. Each nucleotide is color coded.

Porcine fetal fibroblasts in early passage were nucleofected with plasmids expressing a Cas9:GFP fusion and a sgRNA that targets the *COL1A* locus, along with a 200 nucleotide (nt) single stranded DNA oligonucleotide containing two pseudo attP sites (50 nt each; 100 nt total) and 100 nt overall homology (50 nt on either side of the attP sites). The nucleofected cells were cultured in 10% fetal calf serum (FCS) supplemented DMEM medium in the presence or absence of DNA ligase IV and NHEJ pathway inhibitor, SCR7 (10 µM/mL) at 30 °C for three days or 38.5 °C for one day before sorting. One day after nucleofection, the GFP expressing cells were sorted to yield a single GFP expressing cell/well in a 96-well plate. The sorted cells were cultured in irradiated mouse embryonic feeder conditioned medium (CM) supplemented with 5 ng/mL fibroblast growth factor 2 (FGF2) at 38.5 °C in 5% CO_2_ and 5% O_2_. The colonies that formed following sorting were further sub-cultured in 48-well plate at 38.5 °C until confluence, when the colonies were counted, collected by trypsin, and genomic DNA isolated for genetic screening. As shown in Figure 1A and Table 1, less than 2% (1.7%) of the sorted cells established colonies at 30 °C, and inclusion of SCR7 resulting in greater than doubling of colonies established (4.3%). In the established colonies, approximately 13% of colonies showed a 100 base pair (bp) shift in the product size suggestive of a knockin of two pseudo attP sites in the *COL1A* targeting site (that was verified by DNA sequence analysis of an amplicon of this region). Inclusion of SCR7 at 30 °C resulted in greater than a threefold increase (13% *vs.* 44%) in the percentage of targeted colonies (Table 1). When compared to cultures maintained at 30 °C, incubating the plates at 38.5 °C resulted in a doubling of the percentage of colonies in both the absence and presence of SCR7 (3.7% and 10.4%, respectively) (Figure 1B and Table 1). Interestingly, an increase in the number of colonies at 38.5 °C resulted in a corresponding increase in the number of targeted colonies in the absence of SCR7 (13% *vs.* 57%, respectively), but not in the presence of SCR7 (44% in both).

#### 2.1.2. Comparison of Cas9:GFP/sgRNA Plasmid *versus* Ribonucleoprotein Delivery

As noted above, incubation of plates at 38.5 °C resulted in a greater number of established colonies. In order to compare the targeting efficiencies between plasmid and protein cocktail, porcine somatic cells were nucleofected with Cas9 ribonucleoprotein complexes (targeting the same site) and plated at 38.5 °C with or without SCR7 supplementation. When compared to plasmid delivery, nucleofection of Cas9 ribonucleoprotein complex resulted in a fourfold and 1.5-fold increases in the percentage of established colonies in the absence and presence of SCR7 (3.7% *vs.* 15.4% and 10.4% *vs.* 16.2%), respectively (Figure 1C and Table 1). Even though we found more colonies with the use of Cas9 ribonucleoproteins, the fraction of targeted colonies were not higher than plasmid transfections (31% *vs.* 57% or 44%).

### 2.2. Somatic Cell Nuclear Transfer to Generate Clonal COL1A Targeted Fetuses and Fibroblast Lines

**Figure 2:**
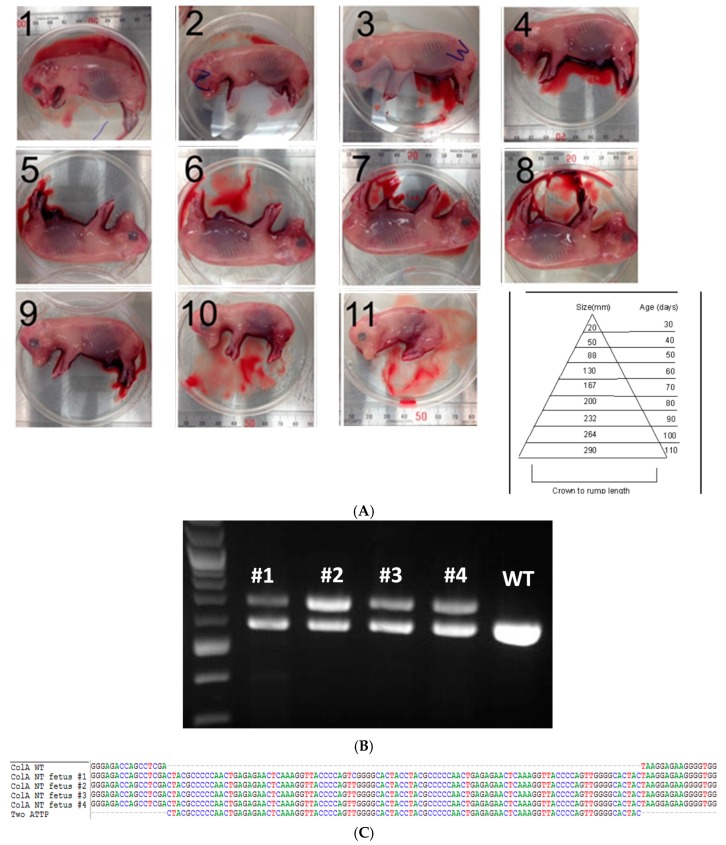
Somatic cell nuclear transfer (SCNT) of porcine *COL1A* targeted fetal fibroblast cells. (**A**) Photomicrograph depicts 11 clonal fetuses (numbered 1–11) at Day 45 of pregnancy along with a chart depicting crown-rump length and age of the fetus measure is shown; (**B**) Agarose gel electrophoresis image of *COL1A* amplicons from fetuses (numbered 1–4) on the top showing targeted knockin of the two attP sites and a 100 bp shift in size. Amplicon from wildtype (WT) animal is shown as a reference; (**C**) Sanger sequencing of the clonal lines confirming the inclusion of the target sites. Multiple sequence alignment of wild type (WT) *COL1A* gene and two pseudo attP knockin sites are shown. Each nucleotide is color coded.

One of the *COL1A* targeted lines generated by CRISPR/Cas knockin and confirmed by sequencing was used as a nuclear donor for SCNT for generating clonal lines. From one embryo transfer session, we have obtained 11 healthy fetuses (Figure 2A). Fetal fibroblast lines were established from four of the 11 fetuses. As expected, PCR-based genotypic analysis revealed that all four fetuses had a 100 bp knockin and shift in size of bands as expected (Figure 2B), which was further confirmed by Sanger sequencing (Figure 2C).

### 2.3. Phic31 Integrase Mediated Integration of Functional GFP Transgene into the attP Modified COL1A Locus

**Figure 3:**
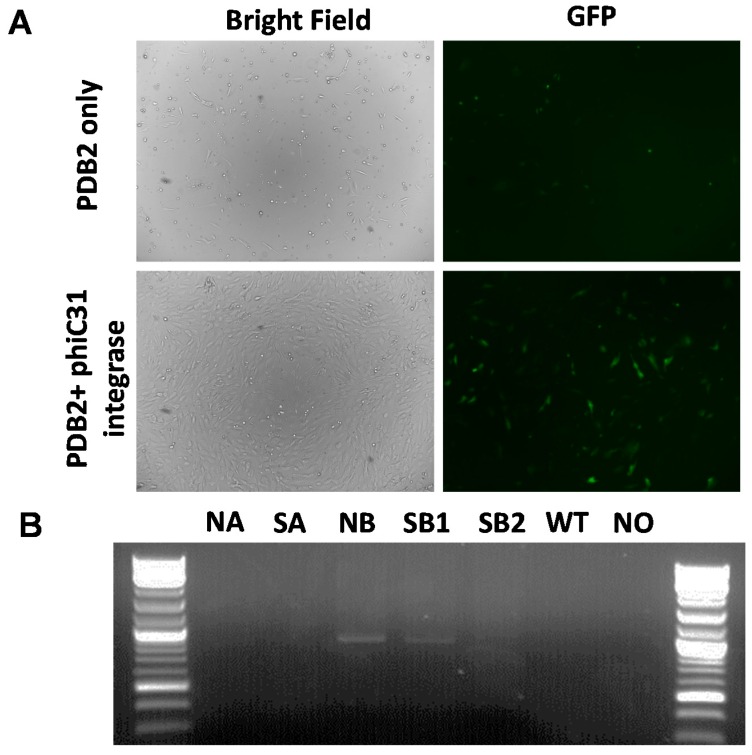
phiC31 integrase mediated integration of GFP transgene into *COL1A* targeted clonal lines. (**A**) Brightfield and fluorescent micrographs (100× magnification) showing integration and expression of GFP transgene in *COL1A* knockin lines in the absence (**top** panel) or presence of integrase (**bottom** panel); (**B**) PCR amplicons showing targeted knockin of GFP transgene into the target sites. The cells following nucleofection and G418 selection and sorting were analyzed by PCR. Amplicons that show knockin were identified only in the presence of integrase and not in vector only treatment, wild type parent cells and no-template controls.

One rationale for introducing attP sites downstream of the *COL1A* gene is to reproducibly and site-specifically introduce functional transgenes into attP sites using phiC31 integrase. A previously published GFP transgene containing phiC31 integrase consensus attB sites and G418 selection cassette, alongside cytomegalovirus promoter driven (CMV)-integrase expressing plasmid was nucleofected into *COL1A* targeted clonal line, and selected for stable integration by G418 selection [22,23]. Stable G418 resistant lines were selected by fluorescence-activated cell sorting (FACS) analysis, which showed integration at the attP sites in the *COL1A* locus (Figure 3). In the future, these sites will form the basis for knockin of other functional transgenes and generation of transgenic animals.

## 3. Discussion

The pig is arguably one of the most valuable animal models for biomedical research as well as for animal biotechnology. That said, the use of pig as a biomedical model is just gaining traction due to widespread and successful adoption of genome editing technologies [8,9,10,11,12,13]. Among the editors, the CRISPR/Cas system has earned the front runner status and greater adoption by the scientific community primarily because of low costs and ease in design, assembly, and delivery. As we show here, sophisticated gene modifications such as knockins, in combination with knockouts and point mutations are easier to achieve in somatic cells by engineering targeted DSBs using CRISPRs.

In this manuscript, we sought to standardize procedures for routine gene targeting and creating a platform for site-specific integration of transgenes using CRISPR/Cas system. First we tested whether transient culture in low temperature promotes HDR as was previously reported [18,19]. In this study, we have not identified any advantage by culturing in low temperatures. There was a greater than twofold reduction in the number of colonies established at 30 °C as compared to 38.5 °C (the temperature at which porcine somatic cells are routinely cultured, presumably due to a lag in cell cycle). The use of SCR7, the inhibitor of NHEJ pathway enhanced colony formation and consequently the efficiency of gene targeting by greater than threefold at 30 °C. Likewise, at 38.5 °C using plasmid transfection, SCR7 increased the number of colonies by three fold; however, the percentage of targeted lines was not different. Clearly, SCR7 solution had a noticeable effect on clonogenicity, however control experiments with just the solvent (DMSO) is lacking to definitively conclude that SCR7 improves clonogenicity. In this manuscript, we also demonstrated that the use of CRISPR ribonucleoproteins is effective, with greater than 1.5-fold increase in the number of colonies established. In the latter case, however, there was no noticeable difference when using SCR7 in either the number of colonies established or the targeting efficiencies. With a noticeable improvement in the number of robustly growing colonies and greater than 30% targeting in the established clones, we argue that CRISPR ribonucleoprotein complex delivery is an effective means for gene targeting experiments. The two main advantages with this approach are: (1) high clonogenicity, requiring seeding and culture in fewer plates potentially making it less labor intensive; and (2) high reproducibility, as the CRISPR reagents are commercially available avoiding differences in reagent preparation between individual investigators. Additionally, with the availability of pseudo attP knockin fibroblast lines, we created a platform for stable integration of transgenes and generation of transgenic animals. Future investigations will be aimed at fine tuning the concentrations of attB containing donor vector and integrase plasmids, sorting the cells into single cells, and identify the percentage of integration of transgene into the attP knockin sites. We believe that this work represents an important step forward in achieving routine targeted genetic modifications in pigs.

## 4. Materials and Methods

All chemicals were obtained from Sigma Chemical Company (St. Louis, MO, USA) unless stated otherwise.

### 4.1. Plasmid Construction and Production of sgRNA

Expression plasmid for Cas9 nuclease (pMJ920) was a gift from Jennifer Doudna (Addgene plasmid #42234) [24]. Targeting guide RNAs were designed based on the software available from MIT (Available online: http://www.genome-engineering.org/crispr/). Two complementary sgRNA oligo DNAs (22 nucleotides in length) were commercially synthesized (IDT DNA Technologies, Coralville, IA, USA), annealed to form double-strand DNA and cloned into a Bsa1 restriction enzyme digested in-house vector to yield a U6 promoter driven sgRNA expression cassette. The cloned fragments were DNA sequenced to confirm their fidelity. Confirmed sgRNA expressing vector was *in vitro* transcribed using MEGAshortscript T7 kit (Life Technologies, Carlsbad, CA, USA) to generate chimeric sgRNA. sgRNA was purified with MEGAclear kit (Life Technologies, CA, USA) for complexing with Cas9 protein prior to nucleofection.

#### Nucleofection and Knockin Experiments

Cas9 plasmid + sgRNA DNA + oligo or Cas9 protein + sgRNA mRNA + oligo were prepared to a final volume of less than 5 μL. The Cas9 protein mixture was incubated for 10 min at room temperature to allow ribonucleoprotein complex formation as per manufacturer’s recommendation (Thermo Fisher Scientific, Waltham, MA, USA). Approximately, 1 × 10^6^ cultured fetal fibroblast cells (culture conditions described below as for sorted cells) were harvested, washed once in PBS, and resuspended in nucleofection buffer (Lonza, Basel, Switzerland). About 5 μL of Cas9 plasmid mixture or Cas9 protein mixture, and cell suspension were combined in a Lonza 4D strip nucleocuvette. Reaction mixtures were electroporated using DO113 setting and immediately plated into one well of a 6-well plate at high density to facilitate recovery. Electroporated cells were incubated (with 10 µM/mL SCR7 or without SCR7) 30 °C for 72 h or 38.5 °C for 18 hand disassociated with 0.05% trypsin for sorting.

### 4.2. Single Cell Sorting and Culture For Colony Screening

Nucleofected cells were sorted for GFP expression on a BD Flowcytometer (San Jose, CA, USA) at the University of Maryland Flowcytometry Core at 1 cell/well density into 96-well plates, which were gelatinized by adding 100 µL of 0.1% gelatin solution for 1–3 h to facilitate attachment. Fifty microliters of 40% FCS High Glucose DMEM which was conditioned (CM) by incubating 20 mL with 2.5 × 10^6^ irradiated CF1 mouse embryonic feeder cells/T75 flask. The CM was supplemented with 5 ng/µL bFGF and filtered before adding to 96 well plate. All wells were fed with 50 µL 10% FCS CM + bFGF immediately after sort, and 50 µL 20% FCS CM after 18 h. The sorted plates were incubated in 5% CO_2_ + 5% O_2_ 38.5 °C for 7–10 days. Colonies that were 80%–100% confluent were split with 0.05% Trypsin, with one well split into 2 wells of 48 well (1:4; this is passage (P1)) and further incubated at 38.5 °C for 3–5 days when 1 well was collected for DNA and the 2nd split into 1 well of 12 well (1:4) for further propagation (P2) or frozen in 92% FCS and 8% DMSO.

### 4.3. Somatic Cell Nuclear Transfer (SCNT)

All animal work was performed as per the approved guidelines of Beltsville ARS, Institutional Animal Care and Use Committee. SCNT was performed as described in previous study [25]. Cumulus-oocyte complexes (COCs) were purchased from a commercial supplier (De Soto Biosciences, Seymour, TN, USA). Briefly, matured oocytes were enucleated by aspirating the polar body and MII chromosomes with an enucleation pipette (Humagen, Charlottesville, VA, USA). After enucleation, a donor cell was introduced into the perivitelline space of an enucleated oocyte. Fusion of injected oocytes was induced by DC pulse (2.0 kV/cm for 30 µs using a BTX-Cell Manipulator 2001 (BTX, San Diego, CA, USA)). After fusion, the reconstructed oocytes were activated by an electric pulse (1.0 kV/cm for 60 µs), followed by 4 h of incubation in PZM3 medium containing 2 mM 6-dimethylaminopurine. Approximately 120–130 reconstructed oocytes were surgically transferred into the oviducts of naturally cycling gilts on the first day of standing estrus. Following transfer, pregnancies were confirmed on Day 30 by ultrasound. Fetuses were harvested from Day 45 pregnant euthanized sow. Tissues from four of the 11 fetuses were trypsin digested and incubated in T175 plates (P0) for subsequent culture and cryostorage.

### 4.4. Genotyping of Fibroblast Cell Colonies, and Edited Nuclear Transfer Fetuses

Single cell derived colonies cultured for 10–15 days were washed three times with PBS-PVA (pH 7.4) medium. About 2–3 µL of colony suspension was transferred into 18 µL of colony lysis buffer (50 mM KCl, 1.5 mM MgCl_2_, 10 mM Tris pH 8.0, 0.5% NP-40, 0.5% Tween-20 and 100 µg/mL proteinase K) and incubated for 1 h at 65 °C. The digestion was terminated by heating the mixture at 95 °C for 10 min, and 2 µL of supernatant was used as a PCR template. Tissue biopsies from fetuses were digested in a tissue lysis buffer (50 mM Tris pH 8.0, 0.1 M NaCl, 20 mM EDTA, 1% SDS, 50 µg/mL RNase A, 100 µg/mL proteinase K) overnight at 65 °C. Following overnight digest, the genomic DNA of the sample was extracted from the tissue lysate using phenol-chloroform, and recovered by resuspension in 100 µL of 10 mM Tris-HCl, pH 7.4 buffer following ethanol precipitation. Purified genomic DNA was amplified using PCR (primers in Table 2), cloned into PCR2.1 vectors (Thermo Fisher Scientific, Waltham, MA, USA) and transformed into *E. coli* DH5-α maximum competent cells (Thermo Fisher Scientific). Five to ten colonies were picked, cultured, plasmid DNA extracted and sequenced (Macrogen, Rockville, MD, USA). Sequences were aligned by Bio-Edit software (Available online: http://www.mbio.ncsu.edu/BioEdit/bioedit.html) for comparison with wild-type and two attP sites per allele.

### 4.5. Targeted phiC31 Integrase Mediated Integration of GFP Transgene into Pseudo attP Sites in COL1A Locus

pDB2 and pCMV-Integrase plasmids were a gift from Michele Calos (Addgene plasmids #18954 and 18935 respectively) [22,23]. Porcine fetal fibroblasts containing pseudo attP sites downstream of *COL1A* site were nucleofected with a previously published GFP transgene (pDB2; 0.7 µg) containing consensus attB site for phiC31 integrase and CMV promoter driven integrase gene (1.3 µg) [26]. A control nucleofection was performed with GFP transgene (pDB2) without the CMV-Integrase plasmid. Following nucleofection, the cells were selected for stable integration by selecting with 500 ng/µL of G418 for 7–10 days followed by flow cytometry. A PCR screen was performed with transgene specific and flanking *COL1A* sequence to show integration of the plasmid at the target site (primers in Table 2).

## Figures and Tables

**Table 1:** Summary of CRISPR/Cas knockin experiments.

Cas9 & sgRNA Combination	Treatment with SCR7	Initial Culture Temperature	Number of Single Cell Sorted Wells	Number of Colonies Formed	% of Colonies Formed/Sorted Wells	Number of Targeted Colonies	Percentage of Targeted Colonies/Colonies Formed
Plasmid	None	30 °C	960	16	1.70%	2	13%
Plasmid	SCR7	30 °C	960	41	4.30%	18	44%
Plasmid	None	38.5 °C	376	14	3.70%	8	57%
Plasmid	SCR7	38.5 °C	376	39	10.40%	17	44%
Ribonucleoprotein	None	38.5 °C	376	58	15.40%	18	31%
Ribonucleoprotein	SCR7	38.5 °C	376	61	16.20%	19	31%

**Table 2:** Primers used in the manuscript.

Target	Primer	Sequence
Gene Knockin Primers	*COL1A* Forward	AGCCAGGCTGCCTTGTTTG
	*COL1A* Reverse	GCCAACCTCCCCTTTGCACT
pDB2 Integration Primers	*EGFPC*	CATGGTCCTGCTGGAGTTCGTG
	*COL1A* Reverse	AGCCAGGCTGCCTTGTTTG

## Abbreviations

The following abbreviations are used in this manuscript:

CRISPR/Cas	Clustered regulated interspaced short palindromic repeat and CRISPR-associated (Cas) nuclease system
SCNT	Somatic cell nuclear transfer
COL1A	Collagen 1A locus
DSB	Double strand break
